# Deep White Matter in Huntington's Disease

**DOI:** 10.1371/journal.pone.0109676

**Published:** 2014-10-23

**Authors:** Owen Phillips, Ferdinando Squitieri, Cristina Sanchez-Castaneda, Francesca Elifani, Carlo Caltagirone, Umberto Sabatini, Margherita Di Paola

**Affiliations:** 1 Clinical and Behavioural Neurology Dept, IRCCS Santa Lucia Foundation, Rome, Italy; 2 IRCSS Neuromed, Pozzilli, Italy; 3 Radiology Dept, IRCCS Santa Lucia Foundation, Rome, Italy; 4 Neuroscience Dept, University of Rome “Tor Vergata”, Rome, Italy; Inserm U837, France

## Abstract

White matter (WM) abnormalities have already been shown in presymptomatic (Pre-HD) and symptomatic HD subjects using Magnetic Resonance Imaging (MRI). In the present study, we examined the microstructure of the long-range large deep WM tracts by applying two different MRI approaches: Diffusion Tensor Imaging (DTI) -based tractography, and T2*weighted (iron sensitive) imaging. We collected Pre-HD subjects (n = 25), HD patients (n = 25) and healthy control subjects (n = 50). Results revealed increased axial (AD) and radial diffusivity (RD) and iron levels in Pre-HD subjects compared to controls. Fractional anisotropy decreased between the Pre-HD and HD phase and AD/RD increased and although impairment was pervasive in HD, degeneration occurred in a pattern in Pre-HD. Furthermore, iron levels dropped for HD patients. As increased iron levels are associated with remyelination, the data suggests that Pre-HD subjects attempt to repair damaged deep WM years before symptoms occur but this process fails with disease progression.

## Introduction

Huntington's disease (HD) is a neurodegenerative autosomal dominate disorder caused by increased CAG repeats, this leads to increased accumulation of mutant huntingtin, and formation of intranuclear inclusions and eventually to brain damage. Damage has long been thought to start in the striatum (for review see: [1]), however, most areas of the brain show abnormalities. Of the abnormalities found in the HD brain, the changes to white matter in particular have been become a major focus of research.

A model for the white matter changes in HD has been formulated [2], which suggests that the pathogenesis of HD begins with a deleterious effect of the mutant huntingtin on myelin. Indeed mutant huntingtin is not found in higher levels in the neurons that degenerate first, rather the mutated protein is expressed throughout the nervous system and periphery [3]. This implies that its primary impact may not be on neurons. The myelin breakdown is, in turn, associated with increased density of oligodendrocytes in the brain, which are involved in repairing myelin damage. As the oligodendrocytes have the highest iron content, when they increase in number, iron content increases [2].

Abnormalities in white matter cause downstream effects through a complex physiological process that is still being uncovered (see [4] for a summary). In short, oligodendrocytes and myelin abnormalities can slow or stop fast axon transport, which can result in synaptic loss and eventually axonal degeneration in a retrograde, (“Dying back”) fashion [5].

In the last decades in-vivo Magnetic Resonance Imaging (MRI) investigations have revealed the extent of changes to white matter in HD. MRI research has reported extensive white matter volume loss in the whole brain [6], in the frontal lobe [7] and across the total cerebrum [8], as well as changes to white matter microstructure; the motor cortico-striatal circuit [9]–[12], sensorimotor cortex, [10], corpus callosum [11], [13], and across extensive distributed white matter fibers (corpus callosum, superior and inferior longitudinal fasciculi and external capsule) [12]) years before symptoms occur.

The increasing evidence that white matter is negatively impacted in HD even before disease onset (Pre-HD) has led us to investigate the changes in the large white matter tracts in Pre-HD and HD and how these changes were related to the iron content. We first investigated the corpus callosum and found that early callosal white matter demyelination damage characterizes HD in the pre-symptomatic stage [11]. At that clinical stage, the myelin breakdown starts at the level of the early and heavily myelinated callosal fibres [14]. Changes in iron content (reduction) manifest in the early stages of HD, likely indicating a failure of the remyelination processes at that time. Then we focused on the cortical spinal tract, which is the brain's main motor fiber [15]. We found that a likely active repair mechanism (as indicated by increased iron) helps keep the cortical spinal tract at a normal functional level in the presymptomatic stage. However, this repair seems to fail with disease progression and cortical spinal white matter tract damage becomes extensive in HD patients. The damage to the cortical spinal white matter tract appears to occur in a retrograde (“Dying back”) fashion, similar to what happens in striatal and cortical projection neurons [5], [16].

In the present study we were interested in making a step forward, by investigating the whole deep white matter in HD, definable as the large easily identifiable association white matter tracts, i.e. the arcuate fasciculus (AF), the superior longitudinal fasciculus (SLF), the cingulate (Cing), the inferior longitidinal fasciculus (ILF), the inferior occipital fasciculus (IFO), the anterior thalamic radiation (ATR) and the uncinate fasciculus (UF). We studied the deep white matter tracts both as a combined whole (called from now on “total deep white matter”) in order to investigate the disease effect on deep white matter in general and as single tracts (AF, SLF, Cing, ILF, IFO, ATR, and UF; we will refer to them as “individual deep white matte tracts”) in order to identify possible regional variations in the disease process.

With the white matter model in mind, we adopted two different MRI approaches to investigate the deep white matter in HD: Diffusion Tensor Imaging (DTI)-based tractography and T2*weighted imaging.

DTI is the MRI technique most frequently used to study white matter fiber changes. It is a non-invasive technique that uses local water diffusion in the brain tissue. Although the biological determinants of diffusion parameters (fractional anisotropy-FA, axial diffusivity-AD and radial diffusivity-RD) are not yet fully understood (for a discussion on diffusion imaging see [17], it is agreed that this approach is sensitive to microstructural tissue properties. Indeed, it has been used in numerous studies to investigate everything from the effects of age on white matter in healthy subjects [18] to disease effects in Alzheimer's disease [19] and schizophrenia [20].

T2* weighted volumes, which are sensitive to iron/ferritin [21], [22] are useful to investigate the iron content that is associated with the remyelination process [2]. The few studies present in literature show a non-univocal picture of the regional white matter iron content changes in HD, with no iron level differences in the callosal splenium, but decreased iron levels in the frontal lobe white matter [2], the callosal isthmus [14] and the cortical spinal white matter tract [15]. However, no studies to date in HD have combined the sensitivity of DTI tractography to identify the deep white matter in-vivo with T2* weighted volume images. By combining these imaging methods in a large cohort of subjects (25 HD patients, 25 Pre-HD and 50 healthy subjects), we can assess the effect HD has on the deep white matter tissue microstructure (total and individual tracts).

The main aims of our study were the following: 1) to examine total deep white matter microstructure in Pre-HD subjects and HD patients; 2) to investigate whether variations in connectivity parameters (FA, AD, RD) and in iron level within the deep white matter provide evidence for the white matter demyelination and remyelination model in HD; 3) to explore whether white matter microstructural properties vary by individual deep white matter tract in a particular manner.

## Methods

### Subjects

Subject demographics and clinical assessments are outlined in Table 1. HD patients (n = 25) and Pre-HD subjects (n = 25), underwent a genetic test (abnormal CAG repeats ≥36) and were examined clinically by the same neurologist (FS) with expertise in HD. All individuals were assessed using the Unified Huntington's Disease Rating Scale (UHDRS), which includes motor, cognitive, behavioral, and functional subscales [23]. Each section consists of a multistep subscale. The motor section measures eye movements, limb coordination, tongue impersistence and movement disorders (such as rigidity, bradykinesia, dystonia, chorea, and gait disturbances). A higher score means more motor impairment. The cognitive scale mainly evaluates executive function. A higher score means better cognitive performance. The behavioral section investigates the presence of depression, aggressiveness, obsessions/compulsions, delusions/hallucinations and apathy. A higher score means more impairment. The functional assessments include the HD functional capacity scale (HDFCS), the independence scale and a checklist of common daily tasks. All three scales mainly investigate independence in daily life activities. The HDFCS is reported as the total functional capacity (TFC) score (range 0–13) and is the only functional subscale with established psychometric properties (including inter-rater reliability and validity), which are based on radiographic measures of disease progression. Thus, the TFC score is used worldwide to determine patients' HD stage. On the independence scale, the investigator indicates whether the patient can perform the task that evaluates independence level (range 10–100). The checklist (functional assessment) is summed by giving a score of 1 to all “yes” answers (range 0–25). Pre-HD are defined as those subjects whom the suspected clinical diagnosis is confirmed by DNA analysis, which revealed (CAG)(n) expansion into the range characteristic of Huntington disease (>36 or repeats), but who do not have manifested Huntington's disease symptoms yet defined by a total motor score of <5 in the UHDRS and cognitive and behavioral assessment within the normality. The Disease Burden index, a measure of disease severity, was used according to the already described formula (age×[CAG-35.5]), where CAG is the number of CAG repeats [24]. A higher score reflects increased disease severity. The Mini Mental State Examination (MMSE) [25], which measures global cognitive functioning, was administered to Pre-HD subjects and HD patients. A lower score reflects greater impairment.

**Table 1:** Sociodemographic and clinical characteristics of patients and control subjects.

	Pre-HD (n = 25)	HD (n = 25)	Controls (n = 25)	Fisher's Exact Test; F or T Test	df	p
**Characteristics**						
Gender male/female	16/9	14/11	30/20	0.367	2	0.833
Age (years ± SD)	37.44±7.01	47.40±14.53	42.88±12.48	4.349	2	0.012^a^ ††
CAG repetition length	43.28±2.17	46.68±6.80	NA	−2.380	48	0.021^a^ ††
MMSE	27.82±1.24	24.97±3.23	NA	3.682	38	0.001^b^ ††
UHDRS Motor	8.00±9.28	37.22±13.18	NA	−8.695	44	0.001^a^ ††
UHDRS Cognitive	257.80±42.34	142.65±50.35	NA	8.046	41	0.001^b^ ††
UHDRS Behavioural	7.67±7.84	18.39±9.13	NA	−4.160	42	0.001^a^ ††
UHDRS Functional	25±0	17.91±5.68	NA	5.984	44	0.001^b^ ††
TFC	13±0	8.39±2.37	NA	9.329	44	0.001^b^ ††
Independence scale	99.8±1.04	78.04±12.49	NA	8.312	44	0.001^b^ ††
Disease burden	292.3±87.52	458.6±104.75	NA	−6.091	48	0.001^a^ ††

Legend. HD = Huntington's disease; Pre-HD = gene-positive, without motor symptoms; SD = standard deviation; df = degrees of freedom; CAG, trinucleotide repeat number; MMSE = Mini Mental State Examination; UHDRS = Unified Huntington's Disease Rating Scale; TFC = Total Functional Capacity; NA = Not Available;

†† T-student, Bonferroni correction.

a Pre-HD<HD (when referred to a cognitive scale comparison or CAG repetition, higher punctuations mean greater impairment).

b Pre-HD>HD (when referred to a cognitive scale comparison, higher punctuations mean lesser impairment).

*MMSE: Missing data for 5 Pre-HD & 5 HD subjects.

*UHDRS Motor: Missing data for 2 Pre-HD & 2 HD subjects.

*UHDRS Cognitive: Missing data for 5 Pre-HD & 2 HD subjects.

*UHDRS Behavioral: Missing data for 4 Pre-HD & 2 HD subjects.

*UHDRS Functional: Missing data for 2 Pre-HD & 2 HD subjects.

*TFC: Missing data for 2 Pre-HD & 2 HD subjects.

Fifty individually healthy subjects were recruited from the community. Patients in the advanced stages of disease (Stages III and IV) and/or with traumatic brain injury or focal lesions were excluded.

### Ethics Statement

Presymptomatic test, genetic diagnosis and clinical exams were performed at Neurological Research Institute IRCCS Neuromed. All participants had the cognitive capacity to understand the research protocol and gave their oral and written consent. Cognitive capacity to consent was determined the MMSE. No subjects had cognitive impairment (see score on Table 1). In no case was there a surrogate consent procedure consented on the behalf of participants. Consent was obtained according to the Declaration of Helsinki and the Santa Lucia Foundation Research Ethics Committee approved the study.

### MRI Data Acquisition

All MRI data was acquired on a 3T Allegra MRI system (Siemens, Germany) using a birdcage head coil. Scans were collected in a single session, with the following pulse sequences: 1) proton density and T2-weighted double turbo spin echo (SE) acquired in transverse planes (time repetition [TR]: 4500 ms, time echo [TE]: 12 ms, time to inversion [TI]: 112 ms, field of view [FOV]: 230×172 mm, matrix: 320×240, slice thickness: 5 mm, number of slices: 24); 2) fluid-attenuated inversion recovery in the same planes as the SE sequence (TR/TE/TI: 8500/109/2000 ms; FOV: 230×168 mm, matrix: 256×256, slice thickness: 5 mm, number of slices: 24); 3) T1-weighted 3D images, with partitions acquired in the sagittal plane, using a modified driven equilibrium Fourier transform [26] sequence (TE/TR/TI: 2.4/7.92/910 ms, flip angle: 15°, 1 mm3 isotropic voxels); and 4) diffusion-weighted volumes were also acquired using SE echo-planar imaging (TE/TR: 89/8500 ms, bandwidth: 2126 Hz/voxel, matrix: 128×128, 80 axial slices, voxel size: 1.8×1.8×1.8 mm) with 30 isotropically distributed orientations for the diffusion sensitizing gradients at a b value of 1000 s/mm2 and 6 b = 0 images. Scanning was repeated 3 times to increase the signal-to-noise ratio.

Six consecutive T2*-weighted gradient echo-planar whole-brain volumes were acquired at different time of echo (TE) (TEs: 6, 12, 20, 30, 45 and 60 ms; TR = 5000; bandwidth = 1116 Hz/vx; matrix size 128×128×80; flip angle 90°; voxel size of 1.5×1.5×2 mm3).

Images were visually inspected for gross anatomical abnormalities by 2 experienced observers (a neuropsychologist expert in neuroimaging and a neuroradiologist) blind to participant identities.

Images were also visually inspected for movement artifacts, which are a common source of concern while studying HD. Since movement can compromise tracking, we excluded subjects who had excessive movement in their scans.

### DTI Processing

Diffusion-weighted images were processed with FMRIB's Software Library (FSL 4.1 www.fmrib.ox.ac.uk/fsl/). Images were corrected for eddy current distortion. The non-diffusion-weighted images were skull stripped using FSL's Brain Extraction Tool (BET) (http://www.fmrib.ox.ac.uk/fsl/bet2/index.html), and used to mask all diffusion-weighted images. A diffusion tensor model was fitted at each voxel using Diffusion Toolkit, generating FA, AD, and RD maps. RD was defined as the average of the second and third eigenvalues of the diffusion tensor, while AD corresponded to the first eigenvalue.

### T2*weighted images processing

T2*weighted volumes were post processed accordingly to previously published methods [21], [27]. Briefly the six T2*weighted volumes were averaged in order to generate a mean T2*-weighted volume. A full affine 3D alignment was calculated between each of the six T2*-weighted volumes and the mean T2*-weighted volume. For each subject we performed a voxel-by-voxel nonlinear least-squares fitting of the data acquired at the six TEs to obtain a mono-exponential signal decay curve. In order to facilitate analysis of relaxation results, we considered the inverse of relaxation times, i.e. relaxation rates R2* = 1/T2*×1000.

Iron images (R2*) were registered into subject DTI space using FSL Flirt 12 degree of freedom transformation with the DTI B0 image used as the reference image. Registrations were visually inspected for accuracy.

### Tractography

Tractography methods are outlined in more detail in [28], however, the tractography and ROI drawing was modified to use TrackVis, an interactive environment for fiber tracking reconstruction, display and analysis developed at the Harvard Medical School Martinos Center for Biomedical Imaging at Massachusetts General Hospital (www.trackvis.org). The FACT approach was used to reconstruct fiber paths. A track angle threshold of 35° was used as well as an image mask based on the B0 image to restrict tracking to biologically plausible results.

Tractography of the total deep white matter was performed by manually drawing regions of interest on each individuals FA color map by a single expert (O.P.) who was blinded to subject age, gender, and diagnosis. To determine intra-rater reliability, fiber tracts were identified in 10 randomly chosen brain volumes. Reliability was assessed using the intraclass correlation coefficient (2-way mixed for intra-rater). The advantage of DTI-based tractography is that the tracts can be precisely mapped within subjects without the reliance on registration prodedures to align imaging data across subjects [28].

Region of interest placement for the AF, SLF, Cing, ILF, IFO, ATR, and UF was based on Wakana, et al [28]. The total deep white matter was for the present study considered to be the combined white matter of the individual tracts (AF, SLF, Cing, ILF, IFO, ATR, and UF). FA, AD, RD, and iron were calculated for each subject by averaging all voxels over the total deep white matter and separately for each individual tract, counting each voxel only once. Excellent intra-reliability was achieved for ROI placement, as determined by computing the intra-class correlation coefficients for tract volume and mean FA, AD, RD and Iron (Table S1). It is important to note, that the specific anatomical delineations for the deep white matter tracts are still emerging. For example, the ILF in [28], may include the middle longitudinal fasciculus (see [29] and the specific delineations of the SLF and AF (temporal component of the SLF) has been a source on contention, [30].

### Statistical Analysis

Demographic differences were assessed using chi-square, independent sample t-tests or Anova as appropriate. All statistical analyses were performed using SPSS 14.0.

To test for total deep white matter differences among groups, a Multivariate Analysis of Variance (Manova) was applied to all three groups (healthy controls, HD patients and Pre-HD subjects). Sex and age were included as covariates in the model. After that, contrasts were run to individuate the significant difference between groups.

In order to localize differences in individual deep white matter tracts between groups, a Multivariate Analysis of Variance (Manova) was applied to all three groups (healthy controls, HD patients and Pre-HD subjects). Sex and age were included as covariates in the model. After that, contrasts were run to individuate the significant difference between groups.

All results were corrected using a False Discovery Rate (FDR) correction, where order ranked p-values less than the resulting q-value were considered significant. Practically, results with a p-value less than 0.03 were considered significant.

## Results

*Subject demographics* are reported in Table 1. Pre-HD subjects and HD patients differed in age and CAG repetition length, but not in gender. Additionally, as expected, HD patients had significantly poorer performances with respect to all measures assessed by the UHDRS, and also a significantly higher score of Disease Burden.

### Total Deep White Matter Findings

**Figure 1:**
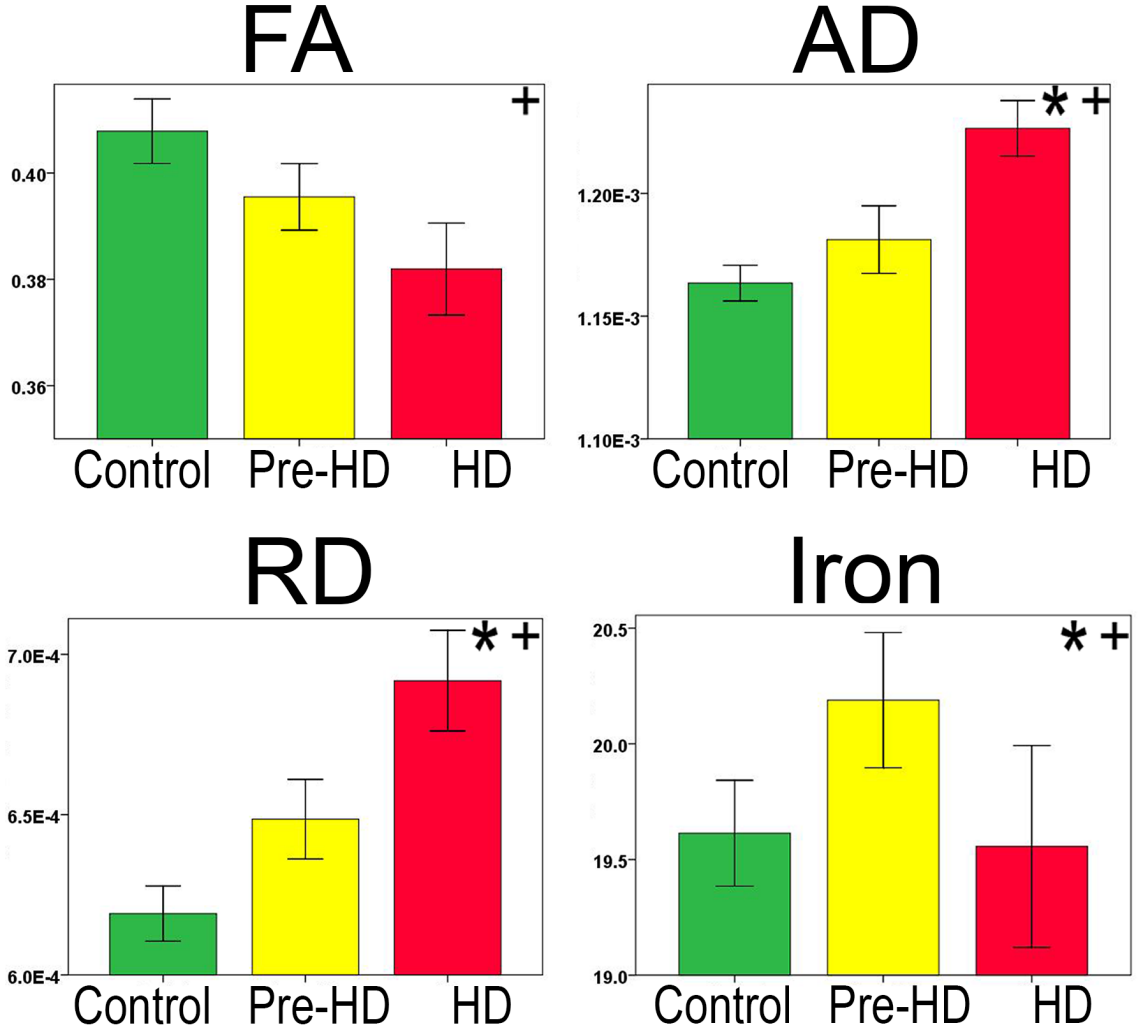
Total Deep White Matter Tractography and Group Comparison. Bar graphs show differences between Total Deep White Matter FA, AD, RD, and Iron. The error bars represent the Standard Error Mean (SEM). Legend. FA = Fractional Anisotropy; AD = Axial Diffusivity; RD = Radial Diffusivity. (AD/RD Mean units: 10^−3^ mm^2^/s, R2* (10^−3^ mm^1^/s). * Significant difference between Pre-HD and Controls+ Significant difference between Pre-HD and HD.

Results are shown in Figure 1.

Statistical details are outlined in Table 2.

**Table 2:** Total Deep White Matter Tractography Group Comparisons.

					MANOVA			Control vs Pre-HD	Control vs HD	Pre-HD vs HD
Region		Pre-HD	HD	Control	F	df	P	P	P	P
	FA	0.399±0.017	0.378±0.022	0.408±0.023	14.81	2,95	0.001	0.048	**0.001**	**0.005**
Total Deep White Matter	AD	1.118E-03±3.00E-05	1.234E-03±3.00E-05	1.116E-03±3.00E-05	41.52	2,95	0.001	**0.013**	**0.001**	**0.001**
(mean value ± SD)	RD	6.38E-04±4.00E-0	7.01E-04±4.00E-05	6.19E-04±3.00E-05	42.132	2,95	0.001	**0.002**	**0.001**	**0.001**
	R2*	20.19±0.75	19.54±1.08	19.62±0.71	4.383	2,85	0.011	**0.010**	0.676	**0.010**

Legend. FA = Fractional Anisotropy; AD = Axial Diffusivity; RD = Radial Diffusivity. (AD/RD Mean units: 10^−3^ mm^2^/s, R2* (10^−3^ mm^1^/s).

*Significant FDR corrected results are in **BOLD**.

**FA:** Pre-HD subjects did not have significantly reduced FA. HD patients had reduced FA compared to Pre-HD subjects and controls.

**AD:** Pre-HD subjects had increased AD compared to controls. HD patients had increased AD compared to Pre-HD and control subjects.

**RD:** Pre-HD subjects showed increased RD compared to controls. HD patients also had increased RD compared to Pre-HD subjects and controls.

R2*: R2* values were elevated in Pre-HD subjects both compared to controls and HD patients. HD patients did not have different values of R2* compared to controls.

We explored the individual covariates' effect on white matter parameters, we found that age has an impact on diffusivity differences (AD and RD) between Controls and Pre-HD subjects, that results in the loss of statistical significance. All other results remain significant in the same direction. We did not find any effect of gender on the white matter parameters.

### Hemisphere Deep White Matter

**Figure 2:**
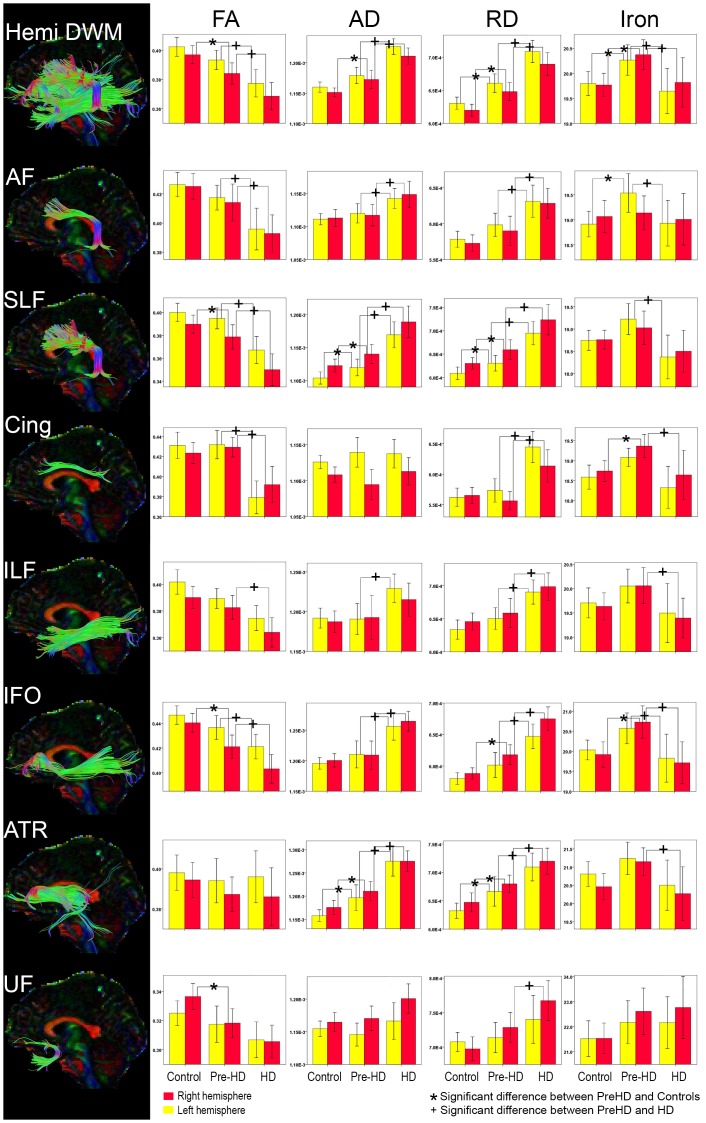
Individual Deep White Matter Tractography and Group Comparison. Bar graphs show differences between Deep White Matter Tracts FA, AD, RD, and Iron. The error bars represent the Standard Error Mean (SEM). Legend. FA = Fractional Anisotropy; AD = Axial Diffusivity; RD = Radial Diffusivity. (AD/RD Mean units: 10^−3^ mm^2^/s, R2* (10^−3^ mm^1^/s) Tracts are from a representative single subject. Legend. Hemi DWM = total Deep White Matter for each hemisphere, it is the result of the individual white matter tracts combined, AF = Arcuate Fasciculus, SLF = Superior Longitudinal Fasciculus, Cing = Cingulate, ILF = Inferior Longitudinal Fasciculus, IFO = Inferior Frontal Occipital fasciculus, ATR = Anterior Thalamic Radiation, UF = Uncinate Fasciculus.

Results are shown in Figure 2.

Hemisphere deep white matter is the combined total of the deep white matter tracts for the left and, separately, for the right hemisphere. Statistical details are outlined in Table 2.

**FA:** Pre-HD subjects had reduced FA in the right hemisphere deep white matter and HD patients had reduced FA in both the left and right hemisphere deep white matter compared to both Pre-HD and control subjects.

**AD:** Pre-HD subjects had increased right hemisphere AD compared to controls. HD patients had increased AD compared to Pre-HD and control subjects in both hemispheres.

**RD:** Pre-HD subjects showed bilateral increases in RD compared to controls and HD patients had elevated bilateral RD compared to Pre-HD subjects and controls.

R2*: R2* values were elevated in both hemispheres within Pre-HD subjects compared to both controls and HD patients. HD patients did not have different values of R2* compared to controls.

### Individual Deep White Matter tract Findings

**Figure 3:**
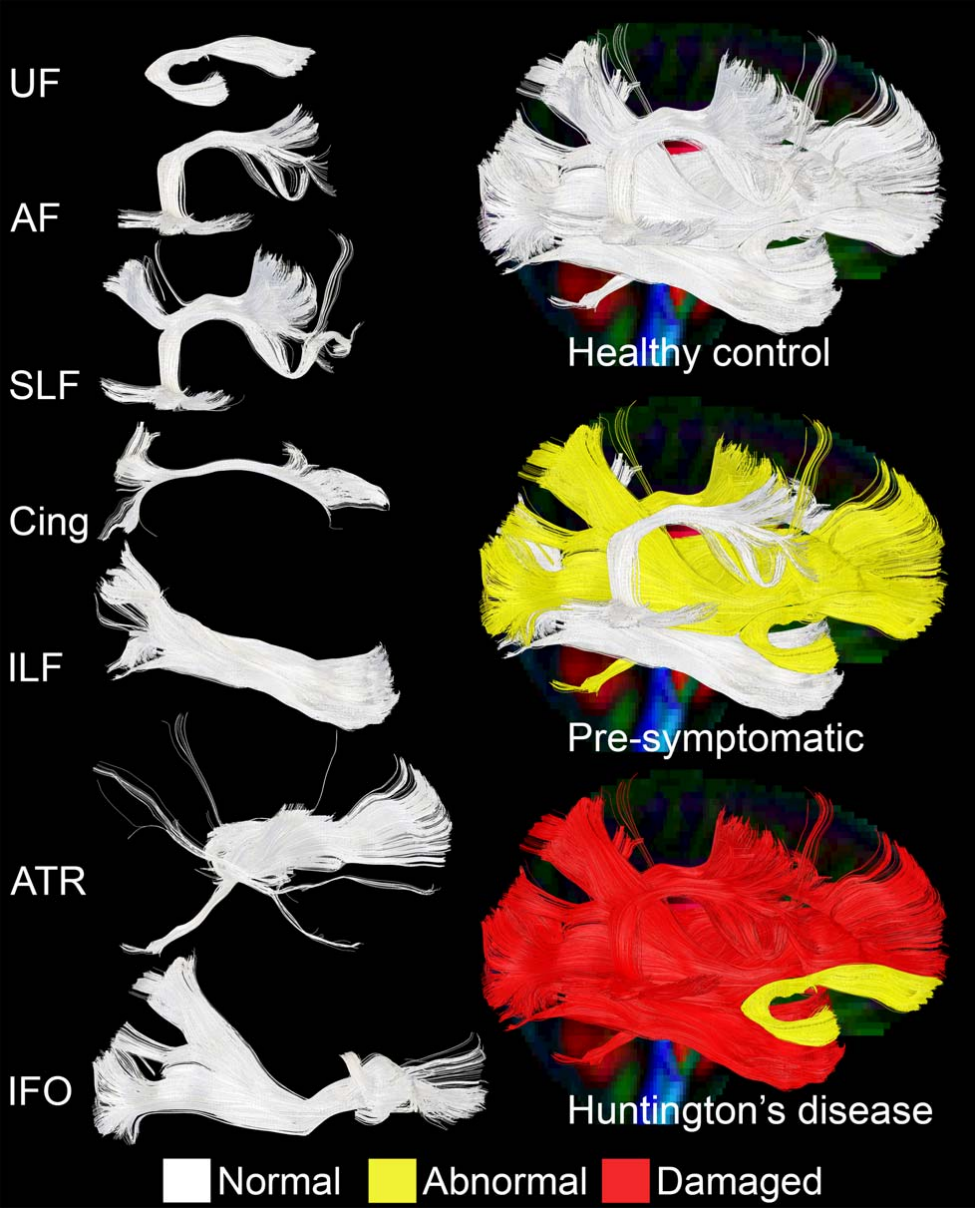
Deep White Matter Microstructure for Each Group. Tracts shown use colour to represent the overall microstructure for each tract within each group. White = Normal, Yellow = Abnormal, Red = Damaged. Tracts Legend. AF = Arcuate Fasciculus, SLF = Superior Longitudinal Fasciculus, Cing = Cingulate, ILF = Inferior Longitudinal Fasciculus, IFO = Inferior Frontal Occipital fasciculus, ATR = Anterior Thalamic Radiation, UF = Uncinate Fasciculus.

Results are shown in Figure 2 and Figure 3.

Statistical details of tractography analysis of the individual deep white matter tracts are in Table 3 and Table S2.

**Table 3:** Individual Deep White Matter Tractography Group Comparisons.

Region	Parameters		MANOVA		Control vs Pre-HD	Control vs HD	Pre-HD vs HD
		F	df	P	P	P	P
	FA	11.72	2,95	0	0.128	**0.001**	**0.007**
Hemi DWM L	AD	41.226	2,95	0	0.033	**0.001**	**0.001**
	RD	42.081	2,95	0	**0.002**	**0.001**	**0.001**
	R2*	3.956	2,85	0.023	**0.027**	0.392	**0.009**
	FA	15.459	2,95	0	**0.025**	**0.001**	**0.008**
Hemi DWM R	AD	32.857	2,95	0	**0.013**	**0.001**	**0.001**
	RD	33.854	2,95	0	**0.004**	**0.001**	**0.001**
	R2*	4.217	2,85	0.018	**0.007**	0.945	**0.023**
	FA	9.489	2,95	0.000	0.284	**0.001**	**0.008**
AF L	AD	7.662	2,95	0.001	0.358	**0.001**	**0.014**
	RD	12.412	2,95	0.001	0.097	**0.001**	**0.007**
	R2*	4.184	2,85	0.018	**0.009**	0.921	**0.018**
	FA	9.259	2,95	0.001	0.204	**0.001**	**0.013**
AF R	AD	5.869	2,95	0.004	0.832	**0.001**	**0.011**
	RD	12.739	2,95	0.001	0.183	**0.001**	**0.003**
	R2*	0.106	2,85	0.899	0.773	0.809	0.647
	FA	10.908	2,95	0	0.132	**0.001**	**0.010**
SLF L	AD	25.772	2,95	0	**0.007**	**0.001**	**0.001**
	RD	26.074	2,95	0	**0.003**	**0.001**	**0.001**
	R2*	5.118	2,85	0.008	0.035	0.133	**0.002**
	FA	14.621	2,95	0	**0.023**	**0.001**	**0.013**
SLF R	AD	18.941	2,95	0	**0.020**	**0.001**	**0.002**
	RD	22.77	2,95	0	**0.002**	**0.001**	**0.004**
	R2*	2.184	2,85	0.119	0.194	0.276	0.040
	FA	11.218	2,95	0	0.453	**0.001**	**0.001**
Cing L	AD	1.282	2,95	0.282	0.131	0.366	0.603
	RD	18.397	2,95	0	0.052	**0.001**	**0.001**
	R2*	2.331	2,85	0.103	0.092	0.499	0.042
	FA	5.607	2,95	0.005	0.852	**0.002**	**0.006**
Cing R	AD	0.573	2,95	0.566	0.343	0.850	0.339
	RD	7.015	2,95	0.001	0.904	**0.001**	**0.003**
	R2*	3.669	2,85	0.03	**0.018**	0.721	**0.019**
	FA	8.587	2,95	0	0.089	**0.001**	0.050
ILF L	AD	7.036	2,95	0.001	0.768	**0.001**	**0.002**
	RD	12.574	2,95	0	0.218	**0.001**	**0.002**
	R2*	1.804	2,85	0.171	0.193	0.395	0.064
	FA	7.896	2,95	0.001	0.337	**0.001**	**0.014**
ILF R	AD	2.333	2,95	0.102	0.764	0.035	0.129
	RD	10.176	2,95	0	0.381	**0.001**	**0.003**
	R2*	3.79	2,85	0.027	0.056	0.235	**0.008**
	FA	8.716	2,95	0	0.146	**0.001**	**0.027**
IFO L	AD	15.865	2,95	0	0.277	**0.001**	**0**
	RD	23.041	2,95	0	0.064	**0.001**	**0**
	R2*	3.781	2,85	0.027	0.050	0.262	**0.008**
	FA	17.85	2,95	0	**0.006**	**0.001**	**0.014**
IFO R	AD	19.794	2,95	0	0.595	**0.001**	**0**
	RD	47.271	2,95	0	**0.005**	**0.001**	**0**
	R2*	7.113	2,85	0.001	**0.003**	0.327	**0.001**
	FA	0.148	2,95	0.862	0.593	0.803	0.810
ATR L	AD	32.937	2,95	0	**0.022**	**0.001**	**0.001**
	RD	16.961	2,95	0	**0.022**	**0.001**	**0.005**
	R2*	2.368	2,85	0.100	0.160	0.283	0.033
	FA	0.848	2,95	0.431	0.344	0.263	0.887
ATR R	AD	30.096	2,95	0	**0.019**	**0.001**	**0.001**
	RD	15.608	2,95	0	**0.025**	**0.001**	**0.008**
	R2*	3.577	2,85	0.032	0.032	0.463	**0.014**
	FA	3.174	2,95	0.046	0.352	**0.014**	0.193
UF L	AD	1.214	2,95	0.302	0.413	0.306	0.124
	RD	2.577	2,95	0.081	0.757	**0.027**	0.110
	R2*	0.893	2,85	0.413	0.274	0.282	0.997
	FA	10.228	2,95	0	**0.017**	**0.001**	0.114
UF R	AD	4.421	2,95	0.015	0.753	**0.004**	0.032
	RD	10.889	2,95	0	0.064	**0.001**	**0.024**
	R2*	2.673	2,85	0.075	0.087	0.046	0.785

Legend. AF = Arcuate Fasciculus, SLF = Superior Longitudinal Fasciculus, Cing = Cingulate, ILF = Inferior Longitudinal Fasciculus, IFO = Inferior Frontal Occipital fasciculus, ATR = Anterior Thalamic Radiation, UF = Uncinate Fasciculus; Hemi DWM = total left and right Deep White Matter, it is the result of the individual white matter tracts combined for each hemisphere; FA = Fractional Anisotropy; AD = Axial Diffusivity; RD = Radial Diffusivity; R = Right; L = Left.

*Significant FDR corrected results are in **BOLD**.

**FA:** Pre-HD subjects revealed, compared to controls, lower FA in the right SLF, IFO and UF for Pre-HD subjects. In comparisons to controls, HD patients had lower FA bilaterally in the AF, SLF, Cing, IFO, ILF, and UF. Finally, FA analyses between Pre-HD subjects and HD patients revealed reductions in HD patients bilaterally in the AF, SLF, Cing, IFO as well as the right ILF.

**AD:** Pre-HD subjects exhibited, compared to controls, increased AD bilaterally in the SLF and ATR. AD analysis between HD patients and controls revealed increased AD bilaterally in the AF, SLF, IFO, and ATR as well as the left ILF and right UF. Furthermore, when comparing Pre-HD subjects with HD patients, HD patients had increased AD bilaterally in AF, SLF, IFO, and ATR as well as the left ILF.

**RD:** Pre-HD subjects, compared to healthy controls, showed significant increased RD in the SLF, and ATR bilaterally, as well as the right IFO. HD patients also showed increased RD compared to healthy controls bilaterally in the AF, SLF, Cing, ILF, IFO, ATR, and UF. Finally, Pre-HD subjects had lower RD compared to HD patients bilaterally in the AF, SLF, Cing, ILF, IFO, ATR and right UF.

R2*: The analysis between Pre-HD subjects and controls revealed increased R2* values in Pre-HD subjects in the left AF and right Cing and IFO. There were no differences in R2* values between HD patients and controls. However, when comparing Pre-HD subjects and HD patients, Pre-HD subjects had increased R2* values bilaterally in the IFO as well as in the left AF, SLF and right Cing, ILF and ATR.

## Discussion

This study sought to identify variations in the deep white matter (total and individual tracts) of Pre-HD subjects and HD patients compared to each other and to healthy controls. A number of major findings emerged from the results: (1) increases in AD and RD in total deep white matter are present in Pre-HD subjects before disease onset; (2) Pre-HD subjects show increased total deep white matter iron content (R2* values) compared to both controls and HD patients; (3) changes in individual white matter tracts in Pre-HD are pervasive and become worse as the disease progresses.

Postmortem studies have repeatedly demonstrated the striking myelin breakdown and white matter atrophy in HD [31]. Furthermore, a HD mouse model [32], found thinner myelin sheaths and increased myelin periodicity (that is less compacted the white matter). Also, an increasing number of white matter DTI studies on human subjects have found extensive distributed changes to white matter microstructure both in Pre-HD and HD subjects [9]–[13], [33]–[36]

Our current findings on total deep white matter are in line with these previous works. We found an increase in AD and RD in Pre-HD subjects compared to healthy controls and abnormalities become significantly worse in HD. The precise biological meaning of the DTI parameters is still unclear [17] and caution should be used in interpreting the results. However, AD a measure of how fast diffusion occurs in the preferred direction, has been shown to be sensitive to the number of axons, as well as their coherence [37]. Increased AD is related to white matter axonal atrophy likely associated with Wallerian degeneration [38]. RD is a measure of how fast diffusion occurs in the perpendicular direction. Increased RD is thought to reflect reductions in myelination [39].

These white matter changes can be interpreted as a manifestation of the pathology's evolution. Indeed, our data suggests that the deep white matter incurs early axonal atrophy (increased AD) and myelin damage (increased RD) even before measurable symptoms appear (Pre-HD stage), and this insult to deep white matter increases progressively as the disease progresses (stage I and II). However, it is important to keep in mind the hypothesis that abnormal brain development may contribute to the pathogenesis of HD, as a precursor to the more global neurodegeneration process [40]. Future longitudinal studies are needed to examine if deep white matter changes are already in place in young Pre-HD subjects.

In order to explore the underlying mechanism of deep white matter abnormalities in HD, we examined the iron content. We found a significant increase in iron content within Pre-HD subjects compared to both healthy controls and HD patients. This suggests that a repair mechanism is active in Pre-HD subjects, and it may lead to an increase in oligodendrocytes before disease onset. The data is in line with previous reports on iron levels in Pre-HD subjects. In fact, extremely elevated numbers of oligodendrocytes have been reported years before symptom appearance [41]–[43]. The ability to repair appears to keep the brain working with minimal symptoms early in the disease and may be why white matter damage has not been identified to be a central part of HD. The repair mechanism may mask the downstream effects the damage causes.

Furthermore, because HD patients have substantially impaired total deep white matter compared to Pre-HD subjects, it may indicate that the repair process is failing. There are two likely reasons for this. One, the oligodendrocytes themselves may be abnormal and thus they do not function correctly and/or the accumulation of oligodendrocytes could be helpful at first, but may be harmful once the levels become too high by causing neuronal excitotoxicity and promoting free radical toxicity [2]. Either of these possibilities fit with the idea that the HD brain is continually trying to remyelinate in a losing attempt to compensate for the disease-related myelin loss [2]. In HD, these remyelination processes may successfully compensate during younger years, which usually correspond to the beginning of the pathology or to the years before onset (Pre-HD stage), but eventually begin failing in older years as brain myelin volume continues to grow and the maintenance of this expanding volume becomes increasingly difficult. This is similar to what happens in healthy older individuals [44] and likely explains the decrease in iron content that we found between Pre-HD and HD subjects.

Interestingly, myelin may play a role in producing adenosine triphosphate (ATP) [46], if this is the case, damage to the total deep white matter could have a substantial impact on the brain's available energy, however, future studies will be needed to verify this.

The R2* measurement has some limitations. Indeed it is sensitive to increases in tissue iron, which increases the R2* value [45] but it is not a specific measure of iron. In fact, many other tissue changes, including myelination, calcifications, blood flow, and increased tissue water, can influence the R2* measure.

Although R2* is not an exclusive measure of iron, combined with diffusivity parameters it helps us reach a conclusion regarding the data. This is because as our DTI findings in Pre-HD subjects indicate tissue damage with more water and myelin damage (see changes in diffusivity Figure 1 and 2), both factors should lower the R2* values in our data. However, we still found higher R2* values in Pre-HD group (Figure 2). This suggests that higher R2* values reflect greater iron in our Pre-HD group.

Furthermore, shimming effects were controled for using the protocol outlined in Peran et al. [27].

However, in the future, higher Tesla strengths, such as 7T or above, and phase images (more related to the iron content) or quantitative susceptibility mapping should be investigated to confirm this data.

In order to identify whether the changes in the deep white matter were localized to individual tracts or presented in a pattern, we examined each tract's microstructure. When comparing controls and Pre-HD subjects, we found the ILF tract was spared in Pre-HD subjects (no difference in FA, AD, RD and iron level). We also found that the AF and Cing did not show any microstructure changes (no difference in FA, AD, RD) but they present a higher level of iron unilaterally (left side for the AF, right side for the Cing) which indicates that the repair mechanism is working in these fibers, hiding the microstructural damage. We further found the IFO and UF were damaged unilaterally (decreased right FA, and increased RD for the IFO, decreased right FA for the UF) and increased iron level in the right IFO which indicates that the repair mechanism is at a breaking point, with both high level of iron and microstructural damage detectable. Finally, we found that the SLF and the ATR were the most damaged tracts at the microstructural level (increased of AD and RD bilaterally) and no difference in iron, which indicates that the repair mechanism already failed in those fibers, and the damage progressed to impair both sides of the tracts.

Thus, it seems that individual white matter tracts are impaired in a specific manner in Pre-HD subjects, and it is possible to describe a pattern in the tract damage, going from spared (ILF), to more damaged (SLF and ATR), passing through those only partially damaged (IFO and UF) to those damaged and under repair (AF and Cing).

When we compare Pre-HD subjects with HD patients, the fibers already damaged in the Pre-HD phase, proceed in a peculiar way. The ILF, initially spared in Pre-HD, becomes impaired in HD (increased AD on the left side; increased RD bilaterally; increased iron on the right side); the AF and Cing, initially with a spared microstructure in Pre-HD, show bilateral microstructural changes in HD; the IFO, damaged unilaterally in Pre-HD, becomes impaired bilaterally in HD; the UF remains stable and relatively spared in both Pre-HD and HD; the SLF and the ATR, already damaged in the Pre-HD phase, become increasingly impaired. We found a high level of iron in Pre-HD compared to HD at the level of the SLF and the ATR, which was not present when Pre-HD subjects were compared with controls, which suggests a repair mechanism was active but is no longer working in HD patients.

Thus it seems that in our sample group: the SLF and ATR are fibers involved in the early phase of the pathology (Pre-HD) while the IFO is relatively spared early but worsens quickly unlike the UF which is more spared throughout the course of the pathology.

The difference in the individual white matter tracts damage (i.e. unilateral or bilateral involvement, early or late involvement in the course of the pathology) may be due to their different function and/or structure. As for the function, it seems there is a link between the fibers we have found damaged or spared (as the UF) and some aspects of the disease. For example, the ATR is involved in executive function and planning complex behaviours and working memory and encoding of new stimuli [47]. The SLF, with its three subcomponents SLFI SLFII and SFLII, is involved in regulation of higher aspects of motor behavior, in spatial attention and in gesture [48], [49]. The IFO is involved in the awareness and use of visual information for the purpose of guiding movements and in the preparation and release of reaching-grasping arm movement [50] as well as the execution of language processing [49]. The UF, which is the spared one, is linked with the regulation of auditory stimuli and recognition memory [50] and likely plays a role in the language network [49]. However, despite our growing understanding of the deep white matter tracts, there is still much we do not yet know about their function thus, cautious consideration is warranted before drawing conclusions between the HD symptomology and specific tracts.

As for the structure, there is some evidence that HD might preferentially damage large early myelinating fibers such as those in the isthmus/splenium of the corpus callosum, which connect to the occipital lobe [13], [14], [33]. Large fibers can be advantageous because they encode more information but even a simple doubling in firing rate appears to more than quadruple an axon's energy use [51]. Large early myelinating high caliber fibers might thus be particularly susceptible. However, all the tracts had significantly worse microstructure as the disease progressed, with changes in FA, AD and RD involving the tracts bilaterally. In particular though, across the DTI parameters (FA, AD, RD), the parameter that was most sensitive to disease effects was RD, indeed each individual tract (AF, SLF; Cing, ILF, IFO and UF) presented an increase in RD for HD patients.

Again our data is in line with the accumulating number of DTI studies of Pre-HD and HD subjects that have looked at white matter. For example, a very recent paper showed extensive alterations to white matter in HD that effects many different anatomical regions [12] and others are rapidly uncovering similar findings [9]–[11]. Because these abnormalities in white matter are being reliably found despite different populations and different methodological approaches, it strongly suggests that there may be a common mechanism damaging white matter across the whole brain in HD. This is important because damage to white matter is extremely detrimental to the brain. There are extensive reasons for this and they are covered in detail by Bartzokis [16]). In short, 1) synapses depends on axonal transport for survival and this is disrupted when white matter is damaged; 2) repair is extremely energetically expensive; 3) repaired myelin is not as good (thinner sheaths, increased number of internodes) as the original undamaged one.

## Conclusions

White matter is critical to the human brain's complexity [16] and we have shown that it is damaged in Pre-HD years before symptoms occur in a specific pattern. Furthermore, Pre-HD subjects attempt to repair this damage but fail as the disease progresses. Finally, the data presented supports the conclusion that changes to deep white matter are a central component of HD.

## Supporting Information

Table S1 Intra-rater Reliability Coefficient.
(DOC)

Table S2 Tract Measures.
(DOC)
