# Supplementary material for: Deep White Matter in Huntington's Disease
Source: PLoS One. 2014 Oct 23;9(10):e109676. doi: 10.1371/journal.pone.0109676 (PMC4207674; doi:10.1371/journal.pone.0109676)
Supplement: Table S1 — Intra-rater Reliability Coefficient. (DOC) [file pone.0109676.s001.doc]

**Table S1:** Intra-rater Reliability Coefficient

|  | **Volume** | | **FA** | | **AD** | | **RD** | | **R2*** | |
| --- | --- | --- | --- | --- | --- | --- | --- | --- | --- | --- |
| **Region** | L | R | L | R | L | R | L | R | L | R |
| AF | 0.991 | 0.916 | 0.993 | 0.941 | 0.99 | 0.987 | 0.995 | 0.958 | 0.989 | 0.922 |
| SLF | 0.938 | 0.963 | 0.968 | 0.979 | 0.985 | 0.981 | 0.987 | 0.972 | 0.994 | 0.966 |
| Cing | 0.964 | 0.917 | 0.981 | 0.972 | 0.956 | 0.972 | 0.98 | 0.946 | 0.97 | 0.979 |
| ILF | 0.975 | 0.954 | 0.933 | 0.948 | 0.888 | 0.935 | 0.935 | 0.971 | 0.942 | 0.901 |
| IFO | 0.972 | 0.941 | 0.936 | 0.989 | 0.976 | 0.967 | 0.961 | 0.98 | 0.989 | 0.987 |
| ATR | 0.945 | 0.927 | 0.971 | 0.978 | 0.913 | 0.957 | 0.906 | 0.922 | 0.93 | 0.968 |
| UF | 0.95 | 0.978 | 0.905 | 0.937 | 0.978 | 0.986 | 0.917 | 0.975 | 0.955 | 0.946 |

Legend. AF = Arcuate Fasciculus, SLF = Superior Longitudinal Fasciculus, Cing = Cingulate, ILF = Inferior Longitudinal Fasciculus, IFO = Inferior Frontal Occipital fasciculus, ATR = Anterior Thalamic Radiation, UF = Uncinate Fasciculus; Volume = Tract Volume, FA = Fractional Anisotropy; AD = Axial Diffusivity; RD = Radial Diffusivity; R = Right; L = Left.
