# Supplementary material for: Deep White Matter in Huntington's Disease
Source: PLoS One. 2014 Oct 23;9(10):e109676. doi: 10.1371/journal.pone.0109676 (PMC4207674; doi:10.1371/journal.pone.0109676)
Supplement: Table S2 — Tract Measures. (DOC) [file pone.0109676.s002.doc]

**Table S2:** Tract Measures

|  |  | **FA (mean ± SD)** | | | **AD (mean ± SD)** | | | **RD (mean ± SD)** | | | **R2* (mean ± SD)** | | |
| --- | --- | --- | --- | --- | --- | --- | --- | --- | --- | --- | --- | --- | --- |
| **Tract** | **H** | Controls | Pre-HD | HD | Controls | Pre-HD | HD | Controls | Pre-HD | HD | Controls | Pre-HD | HD |
| Hemi DWM | L | 0.402±0.024 | 0.398±0.018 | 0.373±0.023 | 1.16E-03±3.25E-05 | 1.17E-03±3.59E-05 | 1.24E-03±3.76E-05 | 6.31E-04±3.64E-05 | 6.49E-04±4.17E-05 | 7.20E-04±5.00E-05 | 19.81±0.75 | 20.26±0.77 | 19.64±1.13 |
|  | R | 0.397±0.022 | 0.389±0.020 | 0.364±0.026 | 1.15E-03±2.66E-05 | 1.17E-03±3.74E-05 | 1.22E-03±3.61E-05 | 6.20E-04±3.59E-05 | 6.38E-04±3.62E-05 | 6.99E-04±4.65E-05 | 19.78±0.72 | 20.39±0.75 | 19.80±1.21 |
| AF | L | 0.426±0.031 | 0.421±0.021 | 0.391±0.036 | 1.11E-03±3.07E-05 | 1.10E-03±3.70E-05 | 1.15E-03±3.82E-05 | 5.79E-04±4.25E-05 | 5.89E-04±4.20E-05 | 6.40E-04±5.84E-05 | 18.92±0.81 | 19.54±0.94 | 18.91±1.18 |
|  | R | 0.425±0.033 | 0.420±0.034 | 0.388±0.031 | 1.11E-03±4.48E-05 | 1.12E-03±4.39E-05 | 1.16E-03±5.23E-05 | 5.73E-04±4.34E-05 | 5.79E-04±5.66E-05 | 6.39E-04±5.38E-05 | 19.09±1.07 | 19.09±0.92 | 19.03±1.37 |
| SLF | L | 0.400±0.028 | 0.395±0.022 | 0.367±0.029 | 1.10E-03±3.24E-05 | 1.12E-03±3.06E-05 | 1.17E-03±4.75E-05 | 6.09E-04±4.64E-05 | 6.30E-04±4.05E-05 | 6.95E-04±5.99E-05 | 18.75±0.71 | 19.22±0.85 | 18.37±1.19 |
|  | R | 0.390±0.028 | 0.379±0.026 | 0.350±0.033 | 1.12E-03±3.49E-05 | 1.14E-03±3.51E-05 | 1.19E-03±5.81E-05 | 6.30E-04±4.36E-05 | 6.59E-04±5.12E-05 | 7.24E-04±7.88E-05 | 18.76±0.66 | 19.03±0.91 | 18.50±1.14 |
| Cing | L | 0.431±0.046 | 0.432±0.034 | 0.379±0.040 | 1.  13E-03±3.30E-05 | 1.14E-03±5.01E-05 | 1.14E-03±4.80E-05 | 5.62E-04±5.30E-05 | 5.74E-04±4.67E-05 | 6.45E-04±6.17E-05 | 18.59±0.94 | 19.08±0.55 | 18.33±1.29 |
|  | R | 0.423±0.037 | 0.429±0.024 | 0.392±0.044 | 1.11E-03±3.83E-05 | 1.09E-03±5.12E-05 | 1.11E-03±4.60E-05 | 5.65E-04±4.66E-05 | 5.56E-04±3.63E-05 | 6.14E-04±6.40E-05 | 18.74±0.80 | 19.36±0.70 | 18.64±1.48 |
| ILF | L | 0.401±0.033 | 0.395±0.018 | 0.370±0.028 | 1.19E-03±4.29E-05 | 1.19E-03±4.69E-05 | 1.23E-03±4.68E-05 | 6.34E-04±5.11E-05 | 6.41E-04±3.91E-05 | 6.99E-04±5.40E-05 | 19.72±0.93 | 20.05±0.91 | 19.48±1.54 |
|  | R | 0.390±0.031 | 0.390±0.025 | 0.358±0.033 | 1.19E-03±5.39E-05 | 1.18E-03±6.87E-05 | 1.22E-03±5.42E-05 | 6.46E-04±5.18E-05 | 6.45E-04±5.78E-05 | 7.11E-04±6.36E-05 | 19.65±0.88 | 20.05±0.91 | 19.38±1.05 |
| IFO | L | 0.447±0.028 | 0.442±0.024 | 0.417±0.026 | 1.20E-03±3.75E-05 | 1.20E-03±5.43E-05 | 1.26E-03±5.16E-05 | 5.80E-04±3.87E-05 | 5.91E-04±4.95E-05 | 6.57E-04±4.77E-05 | 20.09±0.80 | 20.51±1.00 | 19.87±1.52 |
|  | R | 0.440±0.028 | 0.427±0.024 | 0.398±0.030 | 1.20E-03±4.33E-05 | 1.20E-03±6.04E-05 | 1.28E-03±4.54E-05 | 5.89E-04±3.84E-05 | 6.05E-04±4.44E-05 | 6.87E-04±5.23E-05 | 19.93±1.01 | 20.71±1.04 | 19.72±1.24 |
| ATR | L | 0.398±0.032 | 0.395±0.027 | 0.395±0.031 | 1.16E-03±4.36E-05 | 1.19E-03±6.99E-05 | 1.28E-03±7.71E-05 | 6.33E-04±4.94E-05 | 6.59E-04±6.78E-05 | 7.16E-04±5.70E-05 | 20.82±1.05 | 21.24±1.11 | 20.48±1.73 |
|  | R | 0.394±0.031 | 0.390±0.022 | 0.384±0.035 | 1.18E-03±5.61E-05 | 1.20E-03±5.76E-05 | 1.29E-03±6.02E-05 | 6.48E-04±6.21E-05 | 6.68E-04±4.65E-05 | 7.31E-04±5.90E-05 | 20.47±1.10 | 21.15±0.94 | 20.26±1.81 |
| UF | L | 0.325±0.033 | 0.322±0.031 | 0.303±0.028 | 1.16E-03±4.39E-05 | 1.14E-03±4.16E-05 | 1.17E-03±6.77E-05 | 7.08E-04±5.69E-05 | 7.04E-04±5.27E-05 | 7.49E-04±8.19E-05 | 21.53±2.22 | 22.20±2.09 | 22.14±2.52 |
|  | R | 0.336±0.031 | 0.321±0.025 | 0.303±0.030 | 1.17E-03±5.63E-05 | 1.16E-03±4.52E-05 | 1.21E-03±5.98E-05 | 6.98E-04±6.30E-05 | 7.20E-04±5.54E-05 | 7.75E-04±7.85E-05 | 21.56±1.92 | 22.73±2.25 | 22.63±3.09 |

Legend. H = Hemisphere; AF = Arcuate Fasciculus, SLF = Superior Longitudinal Fasciculus, Cing = Cingulate, ILF = Inferior Longitudinal Fasciculus, IFO = Inferior Frontal Occipital fasciculus, ATR = Anterior Thalamic Rdiation, UF =

Uncinate Fasciculus; Hemi DWM = Left and right hemisphere total Deep White Matter, it is the result of the individual white matter tracts combined for each hemisphere; Hemi = hemisphere; FA = Fractional Anisotropy; AD = Axial Diffusivity; RD = Radial Diffusivity. (AD/RD Mean units: 10^−3^ mm^2^/s, R2* (10^−3^ mm^1^/s)
